# Fracture Toughness of Hollow Glass Microsphere-Filled Iron Matrix Syntactic Foams

**DOI:** 10.3390/ma13112566

**Published:** 2020-06-04

**Authors:** Dirk Lehmhus, Jörg Weise, Attila Szlancsik, Imre Norbert Orbulov

**Affiliations:** 1 Department of Shaping and Functional Materials, Fraunhofer Institute for Manufacturing Technology and Advanced Materials IFAM, Wiener Strasse 12, 28359 Bremen, Germany; joerg.weise@ifam.fraunhofer.de; 2 Department of Materials Science and Engineering, Budapest University of Technology and Economics, Műegyetem rakpart 3, 1111 Budapest, Hungary; szlancsik@eik.bme.hu (A.S.); orbulov@eik.bme.hu (I.N.O.); 3 MTA–BME Research Group for Composite Technology, Hungarian Academy of Sciences, Budapest University of Technology and Economics, Műegyetem Rakpart 3, 1111 Budapest, Hungary

**Keywords:** metal matrix syntactic foam, composite foam, hollow glass microsphere, fracture toughness, fracture mechanics

## Abstract

In this study, iron-based metal matrix syntactic foam (MMSF) containing hollow glass microspheres as filler was investigated with respect to notch sensitivity aspects. The MMSF was produced by means of metal powder injection molding. The notch sensitivity was studied via (i) elastic-plastic fracture mechanics measurements (determination of R-curves based on three-point bending tests) and (ii) Charpy impact tests. In both cases, the samples were machined with two different (U- and V-shaped) notch geometries. The critical J-integral value was determined for both notch types, which resulted in lower fracture toughness values in the case of the V-shaped notches and thus notch sensitivity of the material. This finding can be connected to the characteristics of the deformation zone and the associated stress concentration at the tip of the machined notches. The results were confirmed by Charpy impact tests showing ~30% higher impact energy in the case of the U-shaped notch. The failure modes were investigated by means of scanning electron microscopy. In contrast to the bulk material, the MMSF showed brittle fracture behavior.

## 1. Introduction

In the broader spectrum of metallic foams as described, e.g., by Lehmhus et al., syntactic foams occupy positions between purely stochastic foams and designed structures [1]. Typically, in these materials, the size, or at least the size distributions, of hollow fillers are deliberately set, while their relative position can range from arbitrary to ordered. Among them, metal matrix syntactic foams (MMSFs) are high-density foams produced with a metallic (Al [2,3,4,5], Mg [6,7,8,9], Zn [2,10,11,12], Ti [13,14,15], or Fe [16,17,18,19,20] based) matrix and various types of porous fillers (hollow spheres [21] or other porous particles [22]) by molten metal infiltration [23,24]. MMSFs can be utilized as high-performance energy absorbers in vehicles [25] and defensive technologies [26], or as lightweight structural elements. In all cases, the toughness of the MMSFs is important, in addition to the widely investigated conventional and non-conventional mechanical properties [27,28]. These mechanical properties have been widely studied in the aforementioned literature. The results include the observation that, as is the case for non-syntactic foams, the tensile strength tends to be (a) lower than the compressive strength and (b) more dependent on the porosity levels. In short, this implies that a strengthening role of the syntactic foams’ fillers is predominantly relevant under compressive loading conditions. This interpretation is supported by investigations of materials with a 316L matrix, for which high sintering temperatures lead to disintegration of the microspheres and thus a structure resembling conventional rather than syntactic foams. Here, compressive strength (defined as stress at 5% total strain) scales with density according to a power law with an exponent of 1.45 under quasistatic conditions [29], whereas Peroni et al. reported an almost constant plateau for the onset of stress for iron matrix syntactic foams of the same type as scrutinized in the present study under otherwise identical conditions [17,18]. In the latter material, the syntactic foam structure is retained, as microspheres remain intact during the lower sintering temperature of the Fe99.7 matrix [20]. The former value of 1.45 is roughly in line with most publications on conventional, closed-cell metallic foams, as well as the general dependency according to Gibson and Ashby, who suggested a value of 1.5 [30]. We can conclude that MMSFs show significant deviations in their response to compressive and tensile loads, respectively, and that, of these, the latter have so far received much less attention in the scientific literature. We furthermore assume that an improved understanding of crack propagation in MMSFs can shed additional light on their tensile response, and thus concentrate our efforts on fracture mechanical investigations. Previous studies in this area are scarce and mostly dedicated to other types of syntactic and conventional foams. An overview follows below.

Chernousov and Chan defined the toughness of MMSFs as the absorbed mechanical energy during compression stopped at initialization of the first crack [31]. McCullough et al. investigated AlMg1Si06 and AlMg1Si10 closed-cell foams with respect to the fracture toughness aspect by an elastic-plastic fracture mechanics approach (J-integral) performed on compact tension (CT) samples. The denser foams showed a higher fracture toughness, while increasing the Si content made the foams more brittle, confirming a distinctive effect of matrix properties. The rigorous fatigue crack initializing step prescribed was found to be negligible: The fracture toughness results were not affected by a lack of the prefatigued crack compared to the simply notched samples [32]. Olurin et al. investigated the notch sensitivity of closed-cell (Alporas and Alcan) foams based on CT and double-edge notched samples. The investigated samples proved to be notch insensitive and exhibited pronounced R-curve behavior [33]. Olurin et al. investigated the stable propagation of cracks in closed-cell foams at 20 Hz. The study found high exponents (20 < m < 25) in the Paris–Erdogan relationship used to describe the linear part in the kinetic diagram of crack propagation [34]. Motz et al. investigated closed-cell Alporas and Alulight foam CT samples. The fracture toughness values were influenced by the cell morphologies (cell edge and wall thickness, inhomogeneities, etc.) [35]. The same foams were also tested for stable crack propagation parameters at 20 Hz, and high Paris–Erdogan exponents were reported (6 < m < 12) [36]. With respect to open-cell foams, Combaz and Mortensen investigated replicated Al foams made by melt infiltration and the subsequent leaching of NaCl particles. Disk-shaped CT samples were tested and evaluated based on the R-curve theory, with the aim of determining the critical J-integral value of the samples [37]. Besides Al alloys, biocompatible Ti alloys were also investigated in terms of fracture mechanics aspects. The foams studied by Kashef et al. were produced by powder metallurgy from 45 µm, 99.9% pure Ti and 500-800 µm, 99.0% pure NH_4_HCO_3_ space holder powders. R-curve-based measurements were performed on the CT samples, and the results showed that the foams with a higher relative density were tougher and failure of the ligament was due to plastic collapse [13]. Stable fatigue crack propagation tests were also performed, and once again, high Paris–Erdogan exponents were found (15 < m < 20) [15].

In contrast, according to the best of the authors’ knowledge, the published results on the toughness and notch sensitivity characteristics of MMSFs are even more limited. The only example found in the literature is that presented in Szlancsik et al. [38], which discusses an Al matrix system. The aim of the present paper is thus to contribute to filling an apparent gap in research by mapping the notch sensitivity of Fe-based MMSFs with hollow glass microsphere fillers (produced by metal injection molding) by measuring the fracture toughness and impact energy of MMSF samples machined with different, U- or V-shaped notches.

## 2. Materials and Methods 

Diafe 5100 grade carbonyl-iron-powder with a particle size of ~4–6 μm (D10: 1.7–2.7 μm, D50: 3.9–5.2 μm, D90: 7.2–9.2 μm, Dr. Fritsch GmbH, Fellbach, Germany) and a chemical composition of min. 97.8 wt % Fe and approximately 0.75–0.90 wt % C was used as the base material to produce bulk samples and MMSFs with 40 vol% of S60HS grade soda-lime borosilicate glass hollow microspheres commercially available from 3M (Saint Paul, MN, USA, more details about the microspheres are given in [17]). The average diameter of the S60HS hollow spheres was 30 μm, accompanied by an average wall thickness of 1.34 μm. Their isostatic compressive or crush strength was 18,000 psi or 124.1 MPa [16]. The samples were produced by metal powder injection molding, as detailed in [16,17,18], resulting in sample densities of 7.4 gcm^−3^ for the bulk and 4.7 gcm^−3^ for the MMSF samples (36.5% density reduction). Metallographic sections of comparable samples, as well as fracture surfaces revealing the internal structure of the material and the distribution of microspheres, have, e.g., been published in [16,17,18]. They confirm that the comparatively mild sintering conditions at temperatures of 900 °C do not compromise the integrity of the glass microspheres, despite the fact that their glass transition temperature is specified as 600 °C.

**Figure 1:**
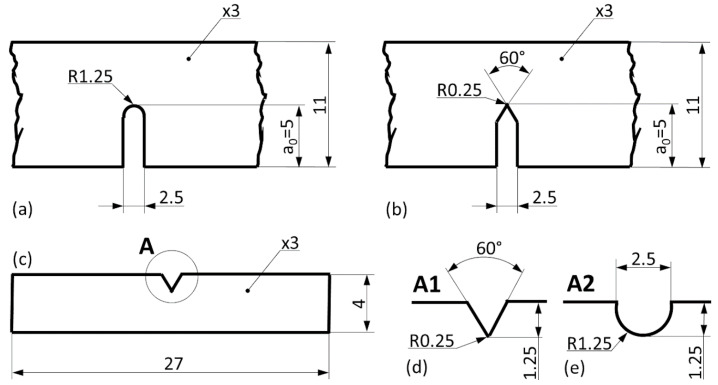
Sample geometries of single-edge notched samples with (**a**) U-notch and (**b**) V-notch; (**c**) sub-sized Charpy sample with (**d**) V-notch and (**e**) U-notch (all dimensions given in mm).

The J-integral values of the materials were determined on single-edge notched three-point bending (TPB) samples. The samples were U- or V-notched to provide an indication of the MMSF material’s notch sensitivity (Figure 1).

The main dimensions of the samples were W = 11 mm (width), B = 3 mm (thickness), and a_0_ = 5 mm. Three samples were tested for each notch geometry and material configuration (12 samples in total). During the tests, the opening of the notch was followed by a double cantilever clip-in displacement gage in the line of the load. The crosshead speed of the tests was 1 mm min^−1^. The tests were performed on an Instron 5965 electro-mechanical testing machine (Instron, Norwood, MA, USA). During the tests, the load was recorded as a function of the notch opening. This method is standardized [39], but the otherwise common use of pre-fatigued notches was neglected based on the findings of McCullough et al. [32]. During the test, the samples were loaded in three-point bending up to the deviation point of the curve from the linear elastic regime. After reaching this point, the load was reduced by a margin of 40% and subsequently reloaded again. The reloading was suspended as soon as a crack opening value of 0.01 mm had been reached. Following this additional crack opening, the loading and unloading cycles were repeated according to [39].

For the Charpy tests, sub-sized standard specimens were employed. The investigations were performed using a Ceast Resil Impactor Junior machine (Instron, Norwood, MA, USA) with a 15 J hammer head (Figure 1c–e). Three samples were tested for each material. Figure 1 shows the dimensions of each sample geometry.

The Finite Element Method (FEM) simulation was applied to qualitatively investigate the strain field in the neighborhood of the notch tips. For this purpose, the strain values in the bulk samples without microspheres were calculated. The sample models were built in MSC Marc Mentat (MSC software, Newport Beach, CA, USA), consisting of 52,870 hexa (8 node cubic) elements. The average element size was 0.5 mm, but the mesh resolution was significantly increased in the area of the notch tips (average element size of 0.02 mm). The applied materials law was elastic-ideally plastic. The elastic modulus was 210 GPa, the yield strength was set to 200 MPa, and the Poisson ratio was fixed at 0.33. The tools were modeled as rigid bodies.

For a closer investigation of the samples’ fracture surfaces, a Zeiss EVO MA10 TYPE electron microscope (Carl Zeiss AG, Oberkochen, Germany) extended by energy dispersive X-ray spectroscopy (EDS) was used. Secondary electron images were recorded to analyze the fracture surfaces. The acceleration voltage was set to 20 kV, and the probe current was 400 pA.

Since the matrix and the filler (and its volume fraction) were identical in all investigated cases, the samples were identified by their notch type (U or V), their number (1 to 3), and their composition (MMSF or bulk).

## 3. Results and Discussion

The MMSFs produced by means of metal powder injection molding had previously been studied in detail regarding their porosity, pore structure, and basic mechanical properties in several studies [16,17,18,20,40,41]. In summary, the materials’ pore size was determined by the dimensions of the 3M^TM^ S60HS hollow glass microspheres, the diameter of which was 30 µm on average at a particle density of 0.6 g/cm^3^, and it thus had a shell thickness of approximately 1.34 µm [18]. The pores, as the microspheres, were spherical in shape, and the compressive strength of the MMSFs was determined at ~200 MPa, depending on the microsphere type and filler content between 5 and 13 wt %. SEM figures and more detailed properties, also for alternative filler materials and matrices, are available in [16,17,18,20,40,41].

### 3.1. Quantitative Results

**Figure 2:**
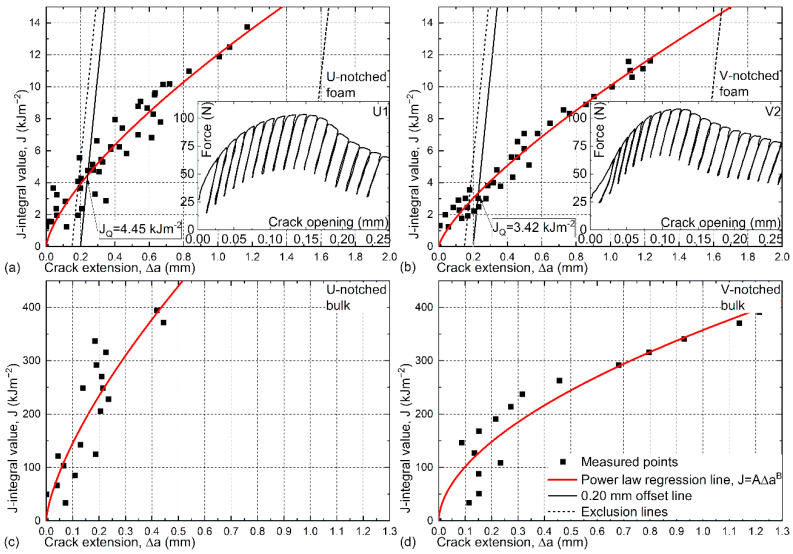
J-integral versus crack extension for U-notched (**a**) foam and (**c**) bulk samples, and V-notched (**b**) foam and (**d**) bulk samples, with representative force–crack opening graphs (sub-figures) for metal matrix syntactic foams (MMSFs).

Regarding the fracture toughness values, the measured force–crack opening curves were evaluated according to the ruling standard on the test method for the measurement of fracture toughness [39]. Since the MMSFs displayed elastic-plastic behavior (see the insets in Figure 2a,b), the J-integral (R-curve) approach was used in the calculations. The measured force–crack opening curves for the foams are plotted as the subfigures of Figure 2a,b and consist of loading and unloading sections, as per the description of the standard. These cycles are used to calculate the crack extension steps (as the ever-increasing maximum crack length during the loading cycles minus the original crack length (a_0_ in our case)) and the corresponding elastic and plastic part of the J-integral value. These calculations are sample shape-dependent. For the given three-point bending curves, the expression of the J integral of the ‘i^th^’ iteration is shown in Equation (1):

(1) $$J_{i}=\frac{F_{\max i\;}{\mathrm{SY}}_{i}\langle \frac{a_{i}}{W}\rangle }{{\mathrm{EBW}}^{3/2}}+\frac{2A_{p\;i}}{B\left(W-a_{i}\right)},$$

where F_max i_ is the maximum force in the i^th^ cycle; S is the span (distance between the supports); Y_i_ is a geometric function depending on the ratio of the actual a_i_ crack length and width (W) of the sample [39]; E is the Young’s modulus of the MMSFs, as derived from the first unloading-loading cycle (the actual individual values are in the range of 8300–9600 MPa); B is the thickness of the samples; and A_p i_ is the plastic part of the recorded curve up to the i^th^ cycle. The upper enveloping curve of the diagrams showed distinct deviations between notch types. In general, the U-notched samples behaved more smoothly, and the radius of the enveloping curve around the force maximum was larger. The sharper V-notch resulted in a smaller radius, representing more brittle behavior. The evaluation of the loading-unloading cycles (as detailed in Equation (1)) led to the representation of the calculated J-integral (J_i_ (kJm^−2^)) values as a function of the crack extension (Δa_i_ (mm)). These are plotted in the main graphs of Figure 2a,b for the MMSFs, and in Figure 2c,d for the bulk matrix material, respectively.

According to the standard, the measured points in Figure 2 must be fitted by a power function curve (Equation (2)):

(2) $$J=A{\left(\Delta a\right)}^{b},$$

where A and b are fitting parameters. Due to the nature of MMSFs, the measured points exhibited relatively large scatter, and due to this, none of the measured points (including the extreme values) could be neglected. Therefore, the R^2^ values of the fittings were relatively low, but still acceptable; namely, 0.91 and 0.93 for the U- and V-notched MMSF samples, respectively. Following the establishment of the fit curve, the graphs for the MMSFs were further detailed by plotting the so-called blunting line (continuous black line in Figure 2a,b) and the corresponding exclusion lines (dashed black line in the figures) [39]. The intersection of blunting lines and fit curves marks the point at which the J-integral values in question (J_Q_) must be read. The evaluation resulted in a J_Q_ value of 4.45 kJm^−2^ in the case of U-notched foam samples and 3.42 kJm^−2^ in the case of V-notched foam samples (U-notched samples performed +30.1% better).

The J_Q_ values must be validated by the inequity system of Equation (3). If all the inequities are true, then the J_Q_ = J_IC_ equation holds, and the J_Q_ values can be understood as valid quantification of the MMSFs’ fracture toughness (J_IC_, a material property).

(3) $$\begin{array}{c} B\\ W-a_{0} \end{array}\}\geq 25\frac{J_{Q}}{\sigma_{F}},$$

where σ_F_ is the so-called ‘flow’ stress of the material, determined as the mathematical average of the proof and the ultimate tensile strength [39]. In previous studies [40], the tensile elongation was found to be small, the proof and tensile strength could not be distinguished, and the tensile strength was taken as σ_F_. Since σ_F_ was relatively high (~107 MPa), the blunting line was steep, and Equation (3) was true for both cases.

Figure 2c,d represent the R-curves of the bulk matrix with the same U- and V-notched shape and under the same test condition. The samples showed extreme (about two magnitudes higher) ductility and toughness values, and according to the standardized method, no quantitative fracture toughness values (as material properties) could be determined from these samples.

In order to further investigate the toughness of MMSF materials, and to facilitate a quantitative comparison with the bulk (matrix) material, further dynamic tests, namely Charpy pendulum tests, were performed. From the Charpy experiments, the impact energies were derived. The results are summarized in Table 1.

The results in Table 1 prove that, under dynamic conditions, the MMSFs show responses similar to the observations made during quasi-static testing. The U-notched samples were able to absorb more energy (+32.6%), while the samples with a smaller notch radius failed at lower impact energies. This observation can be explained by the difference in the affected volume around the tip of the notches. The less concentrated affected volume in the case of the U-notched samples also resulted in higher scatter of the absorbed energies, because the original crack in the samples could be formed in a larger volume and follow other directions (compared to the crack propagation expected based on the opening mode). A larger affected volume implies an increased likelihood of the existence of a local ‘weak link’, e.g., a path along which the microsphere concentration is increased. Besides, the ’non-ideal’ position and propagation of the crack lead to higher impact energies, as well as higher scatter (again due to the higher likelihood of the presence of irregularities in the larger volume) of the measured values.

### 3.2. Qualitative Results

**Figure 3:**
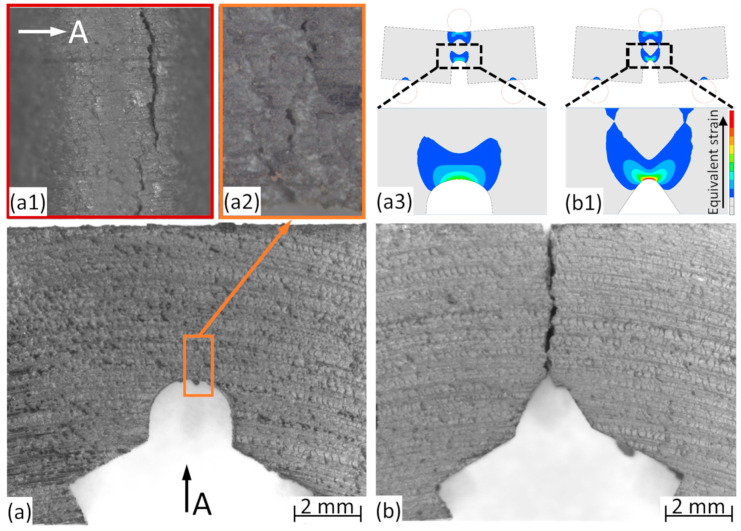
Macrographs of the U-notched MMSF (**a**) and V-notched MMSF (**b**) three-point bending (TPB) samples, including magnified views ((**a1**) and (**a2**) colored boxes) and Finite Element Method (FEM) plots ((**a3**) and (**b1**)**,** results of the simulations performed on bulk samples).

**Figure 4:**
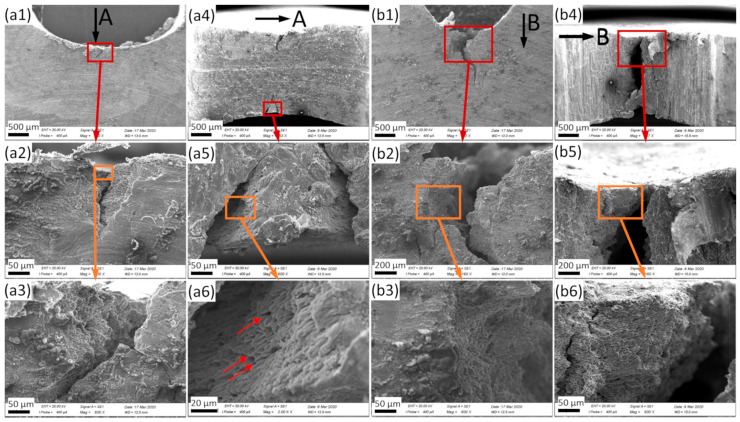
SEM photographs of U-notched (U2) bulk (**a**) and V-notched (V3) bulk (**b**) samples. (**a4**–**a6**) and (**b4**–**b6**) correspond to the views marked by the arrows in (**a1**) and (**b1**), respectively; the location of magnified areas is highlighted by colored rectangles and arrows. Thus (**a1**–**a3**) and (**a4**–**a6**) depict the crack in samples with U-shaped notch from two different perspectives at increasing magnification. (**b1**–**b3**) and (**b4**–**b6**) depict the corresponding images for samples with V-shaped notch.

Examples of cracks and failure modes are shown in Figure 3 for U-notched (Figure 4a) and V-notched (Figure 3b) samples. The reasons behind the differences in the J_Q_ values can be found in the size of the affected material volume around the tip of the machined notch. In the case of the V notch, the deformation is concentrated into a smaller volume. This effect was qualitatively visualized by FEM on bulk samples (Figure 3a3,b1) in terms of the equivalent strain around the notch tips.

**Figure 5:**
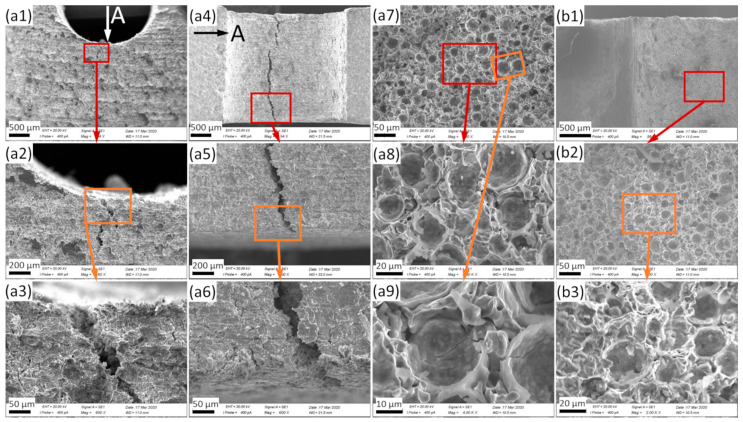
SEM photographs of U-notched (U1) MMSF (**a**) and V-notched (V3) MMSF (**b**) samples. (**a4**) corresponds to the view marked in (**a1**) and vice versa—(**a1**–**a3**) and (**a4**–**a6**) share the same view, respectively; (**a7**–**a9**) and (**b1**–**b3**) are the fracture surfaces of the U-notched and V-notched MMSF samples, respectively. As in Figure 4, the locations of magnified areas are highlighted by colored rectangles and arrows.

The main image in Figure 3a depicts a U-notched MMSF sample. The machining was of a good quality; the contours of the samples are smooth. The crack is poorly visible, since the sample did not fail completely. For this reason, a magnified view is presented in the orange box (Figure 3a2), in which the crack is vertical. The crack was also observed from the direction of the machined notch (marked by view A, red box, Figure 3a1). The crack is not in the centerline of the sample, as can be observed in both magnified views, confirming the larger affected zone in the case of the U-notched samples. In contrast, in Figure 3b, a broken V-notched MMSF sample is shown. The crack origin is at the very tip of the machined notch, as was expected in the case of the sharper notch and reduced size, but more critical affected volume. The failure of the samples was further studied by SEM (Figure 4 and Figure 5).

The samples were investigated in different orientations, depicted here starting with the side view (perpendicular to the plane of the sample: the expected crack propagation direction is downward), followed by observations from the notching tool point of view (i.e., looking into the notch and onto the crack at the notch bottom in the direction of crack propagation, represented by black and white arrows in Figure 4 and Figure 5). In both cases, higher magnification micrographs have also been taken, with the connections between original and subfigures being represented by colored boxes and arrows.

Figure 4 shows the behavior of bulk samples without hollow microspheres. In Figure 4a1, the original site of crack formation in the case of a U-notched (U2) sample can be identified as being located outside the central longitudinal plane of the notch (as shown above). Besides, the crack is more or less straight, with a few wrinkles, and no branching is observed (see Figure 4a2,a3). In Figure 4a4, the lateral contraction can be clearly observed, confirming the ductility and high toughness of the samples. The cracks started from the surfaces. This figure also illustrates that the cracks did not pass through the complete sample, but were arrested within it. Investigating the crack surfaces (Figure 4a6) revealed a ductile fracture with dimples and peaks. Due to the observed changes in the propagation direction of the cracks, a full-area perpendicular view of the fracture surface was not possible. Therefore, the dimples and peaks as depicted here were partly located on planes which were tilted rather than perpendicular to the direction of observation. Some of the peaks are highlighted by red arrows, the direction of which indicates the tilt angle. Figure 4b1 shows the side view of a V-notched (V3) sample. The crack started from the middle of the notch and propagated in the expected direction, with a few turns. In this case, the crack passed through the full sample (Figure 4b4) and the contraction was smaller than in the case of the U-notched sample. This is explained by the higher stress concentration factor of the sharper notch. Nevertheless, in this case, the higher magnification images (Figure 4b2,b3,b5,b6) support ductile fracture of the samples as far as the matrix is concerned.

Figure 5 represents the micrographs taken from the MMSF samples. Figure 5a1 provides a side view of the U-notched MMSF sample U1. The crack was again initiated in an out-of-center position. Crack propagation followed a jagged path according to the actual weakest points in the assembly of the MMSFs in the metal matrix. The same observation can be made in Figure 5a4. The crack line goes through the whole thickness of the sample, but not along a completely straight line. No contraction can be seen in Figure 5a4, and the sample failed in a brittle manner. Regarding the fracture surfaces, the hollow microsphere filler particles can be clearly observed, and the packing density is high (Figure 5a7,a8). The hollow microspheres are broken on the surface, but firmly joined to the matrix, with no empty cavities suggesting a removal of microspheres. In an earlier study [20] on this type of material, differences between the compressive and tensile strength were attributed to a low interface strength, leading to the assumption that this would favor performance under compressive load. Besides, the results of focused ion beam (FIB) investigations, as published in [20] for a similar, though not identical material, have also been interpreted as an indication of a low to no interface strength. The aforementioned results indicate that factors such as the local arrangement of microspheres may be of greater importance. In the present study, the deformation and fracture surface of the matrix between the hollow microspheres shows ductile failure (Figure 5a9), but in general, the sample behaved in a brittle manner, determined by the failure of the hollow glass microspheres. Similar conclusions can be drawn from the SEM micrographs of the V-notched V3 sample. The fracture surface is flat (Figure 5b1) and in general, shows the typical features of brittle failure. At a higher magnification (Figure 5b2), an even and very dense packing of the hollow glass microspheres can be clearly observed. Broken microsphere halves remained in their original position, apparently sticking tightly to the Fe-based matrix material (Figure 5b3). The fact that in fracture surfaces the area proportion of microspheres appears to be larger than the average volume fraction of microspheres supports the assumption that crack propagation follows a weakest path through the sample. This weakest path is, according to all available data on the mechanics of this type of syntactic foam specifically under tensile load, typically linked to the highest porosity, i.e., the highest microsphere content. Within a region of roughly similar global stress levels, as depicted in Figure 3, the crack path will thus selectively follow the highest local microsphere concentrations.

In summary, the investigated MMSFs showed a strong notch sensitivity, and the blunter U-notched samples showed a ~30% higher fracture toughness and ~32% higher impact energy compared to the sharper V-notched samples. This should be considered when any lightweight structural part (please recall the ~36% density reduction) is planned. Consequently, MMSF components should be designed with as few notches as possible for structural applications. The fracture mode of the MMSFs turned to brittle from the original, plastic, and ductile fracture behavior of the bulk samples without hollow microspheres. Considering this, the effect mirrors observations made when subjecting identical materials to conventional tensile testing for the same material (filler and matrix) combination [41]. Based on experiments on Invar matrix materials, which showed significantly higher levels of ductility, Luong et al. [16] have suggested that this effect might be linked to residual stresses introduced during the manufacturing process based on the mismatch in coefficients of the thermal expansion of pure Fe and glass, which is alleviated by using Invar as matrix material. Additional fracture mechanical studies on Invar matrix syntactic foams could thus further elucidate the failure mechanisms in this type of material. Additionally, accompanying high resolution computer tomography studies of the samples tested could allow the role of the weakest link in crack propagation to be verified. Together, such studies could help researchers to understand the relative importance of such possible, competing mechanisms.

## 4. Conclusions

From the analyses and tests detailed presented above, the following conclusions can be drawn:

The fracture toughness of iron-based MMSF filled with hollow glass spheres was successfully measured for the first time;

- Sharper notches resulted in lower critical J-integral values, explained by the smaller affected material volume reaching its critical condition sooner compared to the larger volumes in the case of blunter notches;
- U-notched samples showed a 30.1% higher fracture toughness (J_IC_) and 32.6% higher impact energies under quasi-static and dynamic (impact) conditions, proving a strong notch sensitivity, but independency from the load rate, in the presence of geometrical inhomogeneities (notches);
- Due to the larger affected volumes, the results of the measurements showed larger scatter in the case of U-notched samples;
- The SEM investigations supported the notion of ductile behavior on the part of the bulk samples, as opposed to the brittle fracture of the MMSFs. The fracture surfaces of MMSFs showed broken hollow microspheres, which remained strongly bonded to the Fe matrix.

In summary, MMSF parts for structural applications should be designed with as few notches and geometrical inhomogeneities as possible. Future studies could extend the investigations to alternative matrix materials and accompanying computer tomography for further improved discrimination between the major factors that are assumed to control crack propagation and failure in this type of material.

## Figures and Tables

**Table 1:** Impact energies of the MMSFs.

Sample Number	Impact Energy/KV (J)			
	U Notch		V Notch	
	Bulk	MMSF	Bulk	MMSF
1.	3.28	0.27	2.47	0.21
2.	2.95	0.41	2.35	0.17
3.	3.17	0.23	2.27	0.29
Average	3.13	0.30	2.36	0.22
Scatter	0.17	0.10	0.10	0.06
